# Peculiarities of autoimmune thyroid diseases in children with Turner or Down syndrome: an overview

**DOI:** 10.1186/s13052-015-0146-2

**Published:** 2015-05-15

**Authors:** Tommaso Aversa, Fortunato Lombardo, Mariella Valenzise, Maria Francesca Messina, Concetta Sferlazzas, Giuseppina Salzano, Filippo De Luca, Malgorzata Wasniewska

**Affiliations:** Department of Pediatric, Gynecological, Microbiological and Biomedical Sciences, University of Messina, Via Consolare Valeria, 98125 Messina, Italy

**Keywords:** Chromosomopathies, Course, Epidemiology, Graves’ disease, Hashimoto’s thyroiditis, Pathophysiology

## Abstract

Aim of this commentary is to summarize the salient literature news on the relationships between autoimmune thyroid diseases (ATDs) and either Down syndrome (DS) or Turner syndrome (TS).

According to literature reports both Hashimoto’s thyroiditis (HT) and Graves’ disease (GD) are more frequent in children with DS or TS than in those without these chromosomopathies.

An up-regulation of proinflammatory cytokines might be responsible for the enhanced susceptibility of TS children to ATDs, whereas a dysregulation of immune system may favor the development of ATDs in DS.

In TS children biochemical presentation of HT is less severe than in peer controls. In both DS and TS GD picture at the time of diagnosis is not significantly different than in the pediatric general population.

The evolution over time of GD in DS and TS does not differ from that observed in the pediatric general population, whereas the evolution of HT in both TS and DS is more severe than in girls without these chromosomopathies.

Conclusions: The association with TS or DS is able to affect both epidemiology and course of ATDs by conditioning: a) an increased susceptibility to these disorders; b) a less severe biochemical presentation and a more severe evolutive pattern of HT in TS girls; c) a more severe biochemical presentation and evolution of HT in DS patients.

## Background

Hashimoto’s thyroiditis (HT) and Graves’ disease (GD) are organ-specific autoimmune disorders, that are more common in girls than in boys. In both sexes their frequency increases with age, peaking during adolescence. These autoimmune thyroid diseases (ATDs) occur more frequently in genetically susceptible children, as suggested by the higher concordance in monozygotic than dizygotic twins [1, 2], but their occurrence is also conditioned by environmental triggers, such as iodine intake [3–6].

Other important risk factors for ATDs are familial antecedents of autoimmune disorders, links with other extra-thyroidal autoimmune diseases and association with either Turner syndrome (TS) or Down syndrome (DS), i.e. two common chromosomopathies which are known to be frequently linked with autoimmune illnesses [7, 8].

According to the most recent literature reports, ATDs in children with either TS or DS seem to be characterized by some significant peculiarities in terms of epidemiology, pathophysiology and clinical course. These reports seem to suggest that the association with these chromosomopathies might condition peculiar phenotypical expressions of ATDs [9–14].

Aim of this commentary is to summarize the salient literature news concerning the relationships between ATDs and both these chromosomopathies.

### Epidemiology

In pediatric age HT is a relatively common disease, whose prevalence has been estimated to fluctuate around 1.2 % [15, 16] and is distinctly higher than that reported for GD in the same age bracket, i.e. 1‰ [17] (Table 1).

**Table 1:** Prevalence rates of Hashimoto’s thyroiditis (HT) and Graves’ disease (GD) in the pediatric general population and in young patients with either Turner syndrome (TS) or Down syndrome (DS)

	Pediatric general population	TS	DS
HT	1.2–1.3 % [15, 16]^a^	10–21 % [14, 18–20, 23, 24]^a^	13–34 % [25, 26]^a^
GD	1.07‰ [17]^a^	1.7–3 % [14, 21–23]^a^	6.5‰ [11]^a^

^a^according to the present reference list

In TS girls HT is by far the most common autoimmune disorder and its prevalence has been reported to be even higher than that generally reported in age-matched girls without TS [14, 18–20]. Also the prevalence rate of GD has been repeatedly reported to be distinctly higher in TS girls (from 1.7 to 3.0 %) than in the pediatric general population [13, 14, 21, 22]. Nevertheless, it is very far from that of HT in TS, which is generally reported to range from 10 to 21 % [14, 22, 23] (Table 1).

Also in DS children the prevalence rates of both HT (13–34 %) [24, 25] and GD (6.5‰) [11] are known to be distinctly higher than in the pediatric general population (Table 1).

On overall, on the basis of literature reports, it may be pointed out that both HT and GD are more frequent in the young patients with either TS or DS than in those without these chromosomopathies (Table 1).

### Pathophysiological interpretation of these epidemiological findings

Although it is well assessed, on the basis of the above epidemiological reports, that young patients with TS and DS are more prone to develop ATDs, the pathophysiological mechanisms of these links have not been completely elucidated to date. Nevertheless, in the case of TS children, this association might be, at least partially, explained in the light of the generally recognized links between thyroid autoimmunity and female gender [14]. Another likely and very simple explanation might be that many pediatricians are aware that both TS and DS children are more incline to the risk of developing ATDs and also other autoimmune extra-thyroidal disorders, such as celiac disease, type 1 diabetes, vitiligo and juvenile idiopathic arthritis [19, 26]. Consequently, HT and GD in these conditions might be sometimes suspected by pediatricians even in the presence of minimal thyroid dysfunction symptoms and/or signs [10] and the finding of a thyroid enlargement in these children might be probably considered with more attention. Such an explanation seems to be supported by the observation that ATDs in DS children are generally diagnosed at a younger age than in those without DS [11, 12, 27].

In TS it has been hypothesized that the increased susceptibility to ATDs might be associated with haploinsufficiency of the genes in the pseudoautosomal region of the X-chromosome [26, 28], that may play a relevant part in the pathogenesis of autoimmune diseases [23]. According to other reports, the risk of ATDs is especially high in the patients with X-isochromosome [14, 29, 30], thus suggesting that a gene on the long arm of X-chromosome might be involved in conditioning the predisposition towards ATDs [30]. Other authors, in contrast, have not reported any significant relationships between ATDs and a specific karyotype [13, 22, 31, 32]. Therefore, in the light of the conflicting literature data, no definitive conclusions can be drawn about the influence of karyotype on thyroid autoimmunity in TS.

Other features of TS phenotype, such as an up-regulation of proinflammatory cytokines, might be, at least partially, responsible for the enhanced susceptibility of TS patients to ATDs [33].

In DS the increased prevalence of ATDs has been recently attributed to a dysregulation of immune system [34], with consequent defect of inhibitory activity. These alterations may favor the development of ATDs and other autoimmune disorders [35].

### Clinical and biochemical presentation

Although there are in pediatric literature several studies on the relationships between either TS or DS and ATDs, only few of them have specifically addressed the intriguing issue whether the association with these chromosomal aberrations may in any way condition a different presentation mode of ATDs in pediatric age.

A very peculiar anamnestic feature of GD in both TS and DS children is that its presentation in these conditions is often (20 % of cases) preceded by HT antecedents [9]. It had been already demonstrated that, even in the pediatric general population, there exists a continuum between HT and GD within the spectrum of ATDs [36–40]. The specific observation that children with TS or DS are more likely to progress from HT to GD suggests that the association with these chromosomopathies might favor such a metamorphosis [9].

In DS patients GD does not demonstrate any gender predominance, as against as generally observed in the patients without DS [12]. This finding confirms that children with DS are per se more exposed to the risk of developing GD, irrespective of other concomitant risk factors [12].

In both these chromosomopathies clinical and biochemical picture of GD at the time of diagnosis is not significantly different from that observed in the pediatric general population [12, 13].

In DS children the most common biochemical patterns at HT presentation are subclinical hypothyroidism (SH) and overt hypothyroidism, whereas euthyroidism is distinctly less common than in HT children without DS [41]. In contrast, according to a very recent study, thyroid function impairment at HT presentation seems to be significantly less severe than in girls without TS [10], as substantiated by the higher prevalence of euthyroidism and the lower prevalence rates of both overt hypothyroidism and hyperthyroidism (Table 2). Moreover, in TS girls median TSH serum levels were reported to be significantly lower than in sex-matched peer controls [10]. The less severe impairment of thyroid function at HT presentation in TS children might be explained on the basis of a less aggressive autoimmune pattern [10].

**Table 2:** Prevalence rates (%) of the different thyroid function patterns at Hashimoto’s thyroiditis (HT) presentation in the pediatric general population and in children with Turner syndrome (TS) or Down syndrome (DS), according to our data

	Euthyroidism	Subclinical hypothyroidism	Overt hypothyroidism	Hyperthyroidsm
Pediatric general population^a^	54.3	17.2	22.1	6.4
TS^b^	73.3	23.4	3.3	0
DS^c^	13.7	63.0	19.2	4.1

^a^reference no. [37] of the present list

^b^reference no. [10] of the present list

^c^reference no. [41] of the present list

Table 2 summarizes the prevalence rates of the different thyroid function patterns by us detected at HT presentation in children with TS and DS and in age-matched children without these chromosomopathies.

### Clinical and biochemical evolution over time

Clinical and biochemical evolution under methimazole therapy has been specifically investigated in two studies aiming at ascertaining whether GD children with either DS or TS may exhibit different responses to pharmacological therapy, with respect to young patients without these chromosomopathies [12, 13].

In DS patients the course of GD under methimazole treatment seems to be less severe than in the children without DS [12]. This inference, however, is in contrast with the results of another study on the same issue, according to which the remission rates under pharmacological therapy are very low in DS-related GD and many DS patients with GD may need radioiodine ablation [11].

In TS children both the initial methimazole doses and those needed to maintain euthyroidism during the first treatment cycle were not different than in girls without TS [13]. Also remission rates during the first methimazole cycle, relapse rates after the first cycle withdrawal and cumulative incidences of relapse were reported to not differ than in GD girls without TS. Finally, even the rates of girls who need a non-pharmacological treatment, such as surgery or radioiodine ablation, do not seem to be significantly higher in GD girls with TS [13]. Therefore, according to the only available study aiming at comparing clinical and biochemical responses to therapy in two different series of GD girls with or without TS [13], it may be argued that the evolution over time of GD does not significantly differ in the children with TS than in those without TS.

The spontaneous evolution over time of thyroid function tests in TS children with HT has been only occasionally evaluated so far [10, 14]. According to these reports, such course seems to be characterized by a spontaneous deterioration of thyroid function [13, 14]. In fact, thyroid tests were reported to significantly worsen over time, both in the girls who had presented with euthyroidism and in the ones who had presented with thyroid dysfunctions and this spontaneous trend seems to be irrespective of both karyotype and other factors [10]. Thyroid function deterioration over time seems to be especially evident in the TS girls with initial SH [10]. These findings are particularly striking considering that the natural history of SH in the pediatric population had been so far reported to be generally characterized by a less negative spontaneous trend, both in the cases with an idiopathic form [42–47] and in the ones with an underlying HT [10, 48–50]. To sum up, in the light of these peculiarities which seem to characterize the natural history of TS-related HT, a strict monitoring of thyroid function has to be strongly suggested in these patients [10].

A close monitoring of thyroid tests during follow-up has probably to be suggested also in the children with DS-related HT, at least according to the results of a very recent follow-up study on this specific issue [41]. Moreover, it is well known that the association with DS may sometimes condition a particularly severe clinical expression of autoimmunity [51].

### Influence of the genes affected by TS and DS

There are at least ten genes located on the X-chromosome which are known to be possibly involved in the immuneregulation process [28]. The most important one is FOXP3, that encodes a transcription factor for the function of regulatory T-cells. Its deletion causes immunodysregulation, polyendocrinopathy and X-linked enteropathy [28]. Polymorphisms of this gene are associated with AITDs in Caucasian people [52], thus suggesting that FOXP3 haploinsufficiency might be responsible for the increased risk of AITDs in TS girls [28].

In DS the predisposition to autoimmunity may result from a partial central tolerance failure due to insufficient intrathymic expression of AIRE gene, which is located on chromosome 21 [53]. According to this view, the thymus of DS patients might contain significantly lower levels of AIRE gene, which may in turn condition the predisposition to autoimmunity that is typical of DS [53].

## Conclusions

The association with TS or DS is able to affect both epidemiology and course of AITDs by conditioning: a) an increased susceptibility to these disorders; b) a more frequent shifting from HT to GD; c) a less severe biochemical presentation and a more severe evolutive pattern of HT in TS girls; d) a more severe biochemical presentation and evolution of HT in DS patients.

## Abbreviations

HT: Hashimoto’s thyroiditis
GD: Graves’ disease
ATDs: Autoimmune thyroid diseases
TS: Turner syndrome
DS: Down syndrome
SH: Subclinical hypothyroidism
